# The Interplay of Programmed Cell Death Networks in Cardiovascular Diseases: Mechanisms and Therapeutic Opportunities

**DOI:** 10.31083/RCM50122

**Published:** 2026-07-17

**Authors:** Yuruo He, Xiayu Zhang, Jinhui Duan, Rong Xi, Xiaoli Li, Xiaolin Niu

**Affiliations:** ^1^School of Medicine, Northwest University, 710069 Xi’an, Shaanxi, China; ^2^Department of Cardiology, Tangdu Hospital, The Fourth Military Medical University, 710038 Xi’an, Shaanxi, China; ^3^Department of Cardiology, The Second Affiliated Hospital of Xi’an Jiaotong University, 710114 Xi’an, Shaanxi, China

**Keywords:** cardiovascular diseases, programmed cell death, ferroptosis, apoptosis, necroptosis, autophagy, pyroptosis

## Abstract

Programmed cell death (PCD) encompasses multiple regulated processes, including apoptosis, pyroptosis, ferroptosis, and necroptosis. These pathways form an interconnected network that contributes to the pathogenesis of cardiovascular diseases (CVDs), including atherosclerosis and myocardial ischemia–reperfusion injury. This review highlights cell-type-specific PCD signatures, showing that endothelial cells predominantly undergo pyroptosis and ferroptosis, whereas macrophages exhibit multiple PCD modalities and complex pathological crosstalk. Moreover, the review systematically summarizes key regulatory pathways (e.g., Piezo1 [Piezo-type mechanosensitive ion channel component 1]/NLRP3 [NOD-like receptor family pyrin domain containing 3], and Nrf2 [nuclear factor erythroid 2-related factor 2]/HO-1 [heme oxygenase 1]/GPX4 [glutathione peroxidase 4]), as well as multi-target natural compounds (e.g., melatonin and Guizhitongluo Tablet) that show translational promise and advantages in modulating PCD networks. The review also provides critical insights into major bottlenecks in clinical translation, including nonspecific tissue distribution and the lack of pathway-specific biomarkers. Novel solutions, such as cardiomyocyte-specific delivery systems (e.g., CD47-targeted lipid nanoparticles) and validated biomarkers (prostaglandin-endoperoxide synthase 2 [PTGS2] for ferroptosis), are also proposed. Overall, this review advances our understanding of PCD network regulation in CVDs and proposes innovative precision therapeutic strategies that align with the evolving needs of cardiovascular translational medicine.

## 1. Introduction

Cardiovascular diseases (CVDs) continue to be the leading cause of global mortality and morbidity, claiming approximately 17.9 million lives annually—a figure projected to increase to 23.6 million by 2030 [1]. In addition to their immense socioeconomic burden, the pathophysiological complexity of CVDs—which includes atherosclerosis (AS), myocardial ischemia-reperfusion (I/R) injury, heart failure (HF), and various cardiomyopathies—continues to present major challenges for clinical management [2,3]. Compelling evidence from landmark studies published in Nature and Circulation has firmly established that cardiomyocyte loss and vascular cell dysfunction, driven by regulated cell death, play a central role in both disease initiation and progression [4,5]. In contrast to unregulated necrosis, programmed cell death (PCD) is a genetically regulated process that determines cell fate under stress, rendering it a pivotal therapeutic target for preserving cardiac function and vascular integrity.

Since the initial description of apoptosis in the 1970s, the concept of PCD has undergone profound evolution and significant expansion [6]. For decades, apoptosis was regarded as the sole canonical form of PCD. However, advances in molecular biology and associated technologies have led to the discovery of multiple novel forms of regulated cell death [7]. These include ferroptosis (driven by iron-dependent lipid peroxidation), necroptosis (mediated by receptor-interacting protein kinases), pyroptosis (initiated by inflammasome activation), and autophagy-dependent cell death—each characterized by unique molecular machinery, regulatory networks, and pathophysiological roles [8].

Critically, these PCD pathways do not function in isolation but form a highly interconnected and dynamically balanced signaling network. These pathways frequently share upstream stress signals—such as bursts of reactive oxygen species (ROS), metabolic perturbations, and endoplasmic reticulum stress—and converge with extensive crosstalk at downstream effector levels [9,10]. This paradigm shift has fundamentally reshaped our understanding of CVD pathogenesis, revealing how multiple PCD modalities may act in concert, competition, or antagonism to collectively dictate cell fate and, consequently, disease progression and outcomes.

Against this background, we advance a central hypothesis: complex crosstalk among PCD pathways represents a critical regulatory layer in CVD pathogenesis, and elucidating this network is essential for developing novel targeted therapies. Although individual PCD pathways in CVD have been extensively investigated [11,12], systematic integration of their interactions remains underexplored. Emerging evidence indicates that PCD interplay—such as the apoptosis-to-necroptosis switch or the synergistic interaction between ferroptosis and pyroptosis—critically determines the severity of cardiac injury and the degree of vascular remodeling [13,14]. By synthesizing mechanistic insights from recent studies, this review seeks to dissect the molecular basis of PCD interactions across major CVDs, with particular emphasis on therapeutic vulnerabilities in key pathways. We further underscore that targeting the PCD network as an integrated system, rather than isolated pathways, may provide a more comprehensive and efficacious strategy for mitigating the global burden of cardiovascular disease.

## 2. Core Mechanisms of PCD Subtypes: Classic Pathways and Novel Regulators

Although each PCD subtype is characterized by distinct molecular machinery, these pathways rarely function as isolated modules in CVD. Instead, apoptosis, necroptosis, pyroptosis, ferroptosis, and autophagy are concurrently activated within a common stress milieu shaped by mitochondrial dysfunction, oxidative imbalance, calcium overload, inflammatory signaling, and metabolic perturbations. These shared triggers establish a highly dynamic network wherein distinct PCD programs may coexist, compensate for each other, compete, or shift dominance depending on stress intensity, checkpoint fidelity, and cell-type specificity. Accordingly, the following subsections outline the defining features of each PCD subtype while highlighting their points of convergence and their functional relevance within an integrated cardiovascular death network.

### 2.1 Apoptosis: Canonical Pathway Framework and Novel Crosstalk Regulatory Nodes

Apoptosis constitutes a major form of regulated cell death in CVDs and typically represents the most orderly and controlled cellular response to injury. It facilitates the elimination of damaged cells while minimizing membrane disruption and excessive inflammatory spillover. Apoptosis is executed via two primary pathways: the mitochondrial (intrinsic) pathway and the death receptor (extrinsic) pathway [7].

The intrinsic pathway is initiated by various intracellular stressors, including DNA damage, oxidative stress, metabolic perturbations, and mitochondrial dysfunction. Its regulation primarily hinges on the balance between pro- and anti-apoptotic Bcl-2 family members, especially the interactions of Bcl‑2 homology domain 3 (BH3)-only proteins with Bax, Bak, and other anti-apoptotic Bcl-2 homologs [15]. When the balance shifts toward pro-apoptotic dominance, mitochondrial outer membrane permeabilization (MOMP) ensues, resulting in dissipation of mitochondrial membrane potential, cytochrome c release, and apoptosome assembly via cytochrome c binding to apoptosis protease-activating factor-1 (APAF-1). The apoptosome subsequently activates caspase-9 and downstream executioner caspases (notably caspase-3 and caspase-7) [15,16,17]. In cardiovascular contexts, mitochondrial apoptotic signaling is commonly activated in response to ischemia-reperfusion, diabetic stress, and cardiotoxic insults.

The extrinsic pathway is triggered by the binding of death ligands to their cognate receptors, resulting in the formation of receptor-associated signaling complexes and activation of caspase-8. Activated caspase-8 can either directly cleave and activate executioner caspases or amplify the mitochondrial pathway by cleaving Bid to produce truncated Bid (tBid), which translocates to mitochondria and promotes Bax/Bak oligomerization [18]. This Bid-mediated amplification of mitochondrial damage has been implicated in myocardial ischemia-reperfusion injury and diabetic cardiomyopathy [19].

In CVDs, however, apoptosis does not function as an isolated terminal pathway. Its signaling architecture extensively overlaps with other PCD modalities. For instance, caspase-8 serves not only as an apoptotic initiator but also as a critical checkpoint that suppresses or redirects necroptosis, and in certain contexts contributes to pyroptosis signaling [20]. Similarly, mitochondrial dysfunction and ROS accumulation—key drivers of intrinsic apoptosis—also serve as major upstream triggers for ferroptosis and inflammatory lytic cell death. Mitochondrial quality control thus carries broad significance within the PCD network: efficient clearance of damaged mitochondria attenuates oxidative stress and reduces apoptotic susceptibility, whereas impaired mitochondrial turnover exacerbates multiple death pathways concurrently [21].

Hence, in cardiovascular pathology, apoptosis is best viewed as one branch of a larger cell death decision-making network. Under moderate stress, it typically serves as a contained, non-lytic response; however, under escalating stress, compromised checkpoint control, or heightened inflammatory signaling, the phenotype may shift toward necroptosis or pyroptosis. This transition principle positions apoptosis as a central reference point for understanding PCD network integration, rather than as an entirely independent endpoint.

### 2.2 Necroptosis: Canonical Signaling Cascade and Non-Canonical Pathway Relevance in CVDs

Necroptosis is a regulated lytic form of cell death that integrates the signaling precision of programmed cell death with the membrane-disruptive features of necrosis. In cardiovascular injury, it is increasingly recognized as a major driver of tissue inflammation, cardiomyocyte loss, and adverse ventricular remodeling. The core machinery of necroptosis centers on receptor-interacting protein kinases—principally receptor‑interacting protein kinase 1 (RIPK1) and receptor‑interacting protein kinase 3 (RIPK3)—and the executioner protein mixed lineage kinase domain-like [MLKL].

The canonical necroptotic pathway is initiated by tumor necrosis factor-α (TNF-α) binding to tumor necrosis factor receptor 1 (TNFR1), resulting in the assembly of Complex I [22]. When apoptotic caspase activity is insufficient or inhibited, RIPK1 undergoes deubiquitination and interacts with RIPK3 through RHIM-dependent binding, thereby forming the necrosome. RIPK3 subsequently phosphorylates MLKL, promoting its oligomerization and translocation to the plasma membrane, where it compromises membrane integrity and triggers cell rupture [23]. RIPK3 may further amplify this process by modulating metabolic enzyme activity and enhancing mitochondrial ROS production, establishing a feed-forward loop that exacerbates necroptotic injury [24]. In CVDs, increased levels of phosphorylated RIPK3 and MLKL, together with elevated inflammatory cytokine release, are commonly associated with necroptotic activation.

Recent evidence suggests that cardiovascular necroptosis extends beyond the classical TNF-driven pathway [24]. Alternative upstream activators, including TIR domain-containing adaptor inducing IFN-beta (TRIF)-dependent Toll-like receptor signaling and Z-DNA binding protein 1 (ZBP1)-mediated sensing of aberrant nucleic acids or mitochondrial danger signals, can likewise engage RIPK3-centered death programs [25,26]. Notably, lipopolysaccharide (LPS)—a prototypical Toll-like receptor ligand widely employed to model inflammatory injury—has been shown to induce not only necroptosis but also ferritinophagy-dependent ferroptosis in sepsis-associated cardiac injury [27]. These non-canonical activators are particularly relevant in conditions such as myocardial ischemia-reperfusion injury and heart failure, which are characterized by pronounced sterile inflammation, mitochondrial damage, and redox imbalance. Oxidative modification of RIPK1 and RIPK3 may further promote necrosome stabilization under such conditions [22,24].

Mechanistically, necroptosis is closely interconnected with apoptosis. Although both pathways share upstream receptor-mediated stress signals, their downstream outcomes diverge depending on checkpoint status—particularly caspase-8 activity. When apoptotic execution remains intact, death receptor stimulation predominantly drives apoptosis; however, when this checkpoint fails, the same upstream signals may be redirected toward necroptosis. This apoptosis-to-necroptosis switch exemplifies a fundamental principle of PCD network regulation in CVD: closely related stress signals can elicit distinct cell death phenotypes depending on the molecular context. Furthermore, as necroptosis culminates in membrane rupture and release of intracellular contents, it functionally converges with pyroptosis as an inflammatory lytic cell death modality, despite differences in their proximal machinery. Thus, necroptosis occupies a pivotal interface between failed apoptotic checkpoint control and amplified inflammatory injury.

### 2.3 Autophagy: Bidirectional Regulatory Balance and Novel Modulators in Cardiomyocyte Homeostasis

Autophagy is a highly conserved intracellular degradation process that preserves cellular homeostasis through the recycling of damaged proteins and organelles. In the cardiovascular system, it holds particular significance for cardiomyocytes, which critically depend on mitochondrial integrity and the efficient clearance of stress-damaged cellular components [28,29]. In contrast to apoptosis, necroptosis, pyroptosis, and ferroptosis, autophagy is not invariably a terminal death mechanism; rather, it predominantly functions as a context-dependent modulator that can either promote cell survival or contribute to injury, contingent on the extent and timing of its activation.

The canonical autophagy machinery depends on two ubiquitin-like conjugation systems. In the first, pro-LC3 is sequentially processed by autophagy‑related gene (Atg) 7 and Atg3, leading to its conjugation with phosphatidylethanolamine and formation of LC3-II, which facilitates autophagosome membrane association [30,31]. In the second system, Atg12 is covalently conjugated to Atg5, forming a complex with Atg16 that drives phagophore elongation and autophagosome maturation [30]. Selective mitophagy is especially relevant in CVDs. Upon mitochondrial damage, PINK1 accumulates on the outer mitochondrial membrane and recruits and activates the E3 ubiquitin ligase Parkin, which ubiquitinates outer mitochondrial membrane proteins and recruits autophagic receptors to facilitate sequestration of damaged mitochondria into autophagosomes [28,29,30].

A hallmark of autophagy in the cardiovascular context is its bidirectional role. Basal or moderate autophagic activity promotes cell survival by preserving proteostasis and attenuating ROS accumulation via clearance of dysfunctional organelles, particularly mitochondria [28]. Conversely, impaired autophagic flux allows accumulation of damaged proteins and organelles, thereby exacerbating oxidative and inflammatory stress. Excessive or maladaptive autophagy, in turn, may contribute to autolysis, energy depletion, and accelerated cell loss [31]. Emerging evidence further suggests that non-coding RNA networks, including lncRNAs and circRNAs, finely regulate this balance by modulating autophagy-related proteins and stress-responsive pathways during cardiovascular injury [32].

Within the broader PCD network, autophagy primarily serves as a modulator of cell death pathway selection rather than as an isolated execution pathway. By maintaining mitochondrial quality, redox homeostasis, and substrate availability, autophagy can suppress apoptotic initiation, inhibit inflammasome activation and pyroptosis, or attenuate ferroptosis susceptibility by reducing oxidative burden. Conversely, defective or excessive autophagic flux may predispose cells to these destructive death pathways. Autophagy thus acts as a key network modulator that determines whether a stressed cardiovascular cell adapts, survives transiently, or succumbs to one or more forms of programmed cell death.

In contrast to the survival‑promoting functions of autophagy, pyroptosis represents a pro‑inflammatory and lytic pathway that directly couples stress sensing with membrane disruption.

### 2.4 Pyroptosis: Non-Canonical Activation Mechanisms of Gasdermin Family and Cardiovascular Implications

Pyroptosis is a pro-inflammatory form of regulated cell death characterized by cellular swelling, pore formation in the plasma membrane, membrane rupture, and release of potent inflammatory mediators. In CVDs, it has emerged as a key mechanism that couples innate immune activation to direct tissue injury, particularly in vascular inflammation, atherosclerotic plaques, and ischemic myocardial damage.

In the canonical pathway, inflammasome sensors—including NOD-like receptor (NLR) family members and absent in melanoma 2 (AIM2)—assemble multiprotein complexes that activate caspase-1. Activated caspase-1 cleaves gasdermin D (GSDMD) to produce an N-terminal fragment that oligomerizes and forms pores in the plasma membrane [33,34]. Simultaneously, caspase-1 matures pro-IL-1β and pro-IL-18, thereby linking membrane permeabilization to robust inflammatory signaling. In the non-canonical pathway, human caspase-4 and caspase-5 (and murine caspase-11) directly detect cytosolic lipopolysaccharide and cleave GSDMD, while also promoting secondary NOD-like receptor family pyrin domain containing 3 (NLRP3) activation via potassium efflux [33,34]. Both pathways converge on plasma membrane permeabilization and inflammatory cell lysis.

Pyroptosis is now recognized to extend beyond canonical inflammasome activation. Alternative gasdermin activation mechanisms have been identified under specific stress conditions. For instance, metabolic stressors such as amino acid starvation and ATP depletion can trigger unc‑51‑like autophagy activating kinase 1 (ULK1)-dependent phosphorylation of gasdermin family members, thereby initiating pyroptosis-like membrane disruption [35]. Furthermore, gasdermin-mediated permeabilization is not restricted to the plasma membrane; mitochondrial membrane injury can occur early in pyroptosis, thereby exacerbating ROS production and amplifying inflammatory cell death [13]. These observations are especially pertinent to CVDs, where mitochondrial dysfunction and sterile inflammation frequently coexist.

Functionally, pyroptosis shares key characteristics with necroptosis: both are lytic and pro-inflammatory, and both amplify local tissue injury via release of intracellular danger-associated molecular patterns (DAMPs). Concurrently, pyroptosis intersects with apoptosis and ferroptosis through common upstream triggers, including mitochondrial damage, oxidative stress, calcium dysregulation, and metabolic compromise. This explains why pyroptosis rarely occurs in isolation but typically constitutes part of a broader inflammatory cell death landscape in cardiovascular lesions. Accordingly, pyroptosis is best regarded as a major pro-inflammatory effector pathway that both receives inputs from and reciprocally regulates the broader PCD network.

### 2.5 Ferroptosis: GPX4-Independent Defense Systems and Emerging Regulatory Mechanisms

Ferroptosis is an iron-dependent form of regulated cell death driven by uncontrolled lipid peroxidation. Although mechanistically distinct from caspase-dependent apoptosis and RIPK-mediated necroptosis, ferroptosis in CVDs is extensively integrated with other PCD pathways via common links to mitochondrial dysfunction, redox imbalance, and inflammatory amplification.

The canonical ferroptosis framework is delineated by three interconnected metabolic axes: iron metabolism, cystine/glutamate antiporter system Xc⁻-dependent amino acid metabolism, and phospholipid remodeling [36]. In iron metabolism, transferrin receptor 1 (TFRC)-mediated iron uptake and nuclear receptor coactivator 4 (NCOA4)-dependent ferritinophagy elevate the labile iron pool, thereby driving ROS generation via the Fenton reaction [37]. System Xc⁻, composed of the light subunit SLC7A11 (solute carrier 7A11) and the heavy subunit SLC3A2 (solute carrier 3A2), mediates cystine–glutamate exchange essential for glutathione (GSH) synthesis. GSH is essential for glutathione peroxidase 4 (GPX4)-mediated reduction of lipid peroxides; disruption of this axis depletes cellular antioxidant defenses and sensitizes cells to ferroptosis. In phospholipid remodeling, acyl‑CoA synthetase long‑chain family member 4 (ACSL4) and lysophosphatidylcholine acyltransferase 3 (LPCAT3) incorporate polyunsaturated fatty acids (PUFAs) into membrane phospholipids, rendering them highly vulnerable to lipoxygenase- or ROS-mediated peroxidation [38].

Recent advances have considerably broadened this paradigm beyond sole reliance on GPX4. Complementary antioxidant systems—most notably the ferroptosis suppressor protein 1 (FSP1)–coenzyme Q10 axis and tetrahydrobiopterin-dependent pathways—also suppress lipid peroxidation and modulate ferroptosis sensitivity [38,39,40,41]. Moreover, post-translational modifications (including ubiquitination), subcellular localization, and redox-sensitive regulation of ferroptosis-related proteins further determine whether lipid oxidative stress culminates in cell death [40,42]. These regulatory layers are particularly pertinent to CVDs, where perturbations in iron homeostasis, mitochondrial metabolism, and ROS signaling commonly coexist.

Although ferroptosis is defined by a unique metabolic execution mechanism, its pathological sequelae frequently overlap with those of pyroptosis and necroptosis, particularly in settings dominated by sterile inflammation and oxidative stress. Mitochondrial damage promotes ferroptosis by elevating ROS levels and compromising metabolic resilience, whereas lipid peroxidation products and associated damage signals can reciprocally amplify inflammatory responses. Consequently, in cardiovascular pathology, ferroptosis typically functions less as an isolated pathway and more as a predominant metabolic death effector embedded within a broader stress-response network. This strategic position within the network renders ferroptosis particularly attractive as a therapeutic target, as interventions at redox, iron-handling, or lipid-remodeling nodes can simultaneously modulate multiple downstream injury cascades.

Building upon these molecular foundations, the following section explores the PCD network in specific cardiovascular contexts, highlighting how these interconnected pathways orchestrate disease‑specific injury patterns.

## 3. The PCD Network in Action: Driving Specific Cardiovascular Pathologies

The organization of the PCD network varies substantially across different cardiovascular diseases. In certain conditions, a temporal hierarchy predominates, with distinct death programs prevailing at successive stages of injury. In others, the network coalesces around a central hub, such as mitochondrial dysfunction or redox imbalance. In atherosclerosis, however, the network is predominantly organized according to cell type within the plaque microenvironment. To more effectively capture this integrative framework, the subsequent subsections are structured around four recurring themes: clinical context, dominant PCD node or hub, principal crosstalk axes, and key therapeutic implications or unresolved issues.

### 3.1 The Temporal Regulatory Mechanism of the PCD Network in Myocardial I/R Injury

Myocardial I/R injury remains a primary determinant of clinical outcome following coronary reperfusion in acute myocardial infarction. Although timely reperfusion is critical for myocardial salvage, the sudden reintroduction of oxygen, ionic shifts, inflammatory activation, and mechanical loading trigger a secondary wave of injury that cannot be attributed to any single death pathway. Rather, myocardial I/R injury is increasingly recognized as a dynamically reconfigured PCD network, in which the predominant execution mechanism evolves across different phases of injury.

#### 3.1.1 Early Phase Core: Piezo1‑Mediated PANoptosis Coactivation

During the early phase of I/R injury, Piezo-type mechanosensitive ion channel component 1 (Piezo1)-mediated mechanotransduction serves as a major upstream hub. In ischemia and early reperfusion, cardiomyocytes experience rapid alterations in cell volume, membrane tension, and mechanical load; Piezo1 functions as a mechanosensitive ion channel that transduces these biophysical cues into intracellular death signaling [43]. Downstream of Piezo1, caspase-8 acts as a pivotal execution checkpoint, orchestrating apoptotic, necroptotic, and pyroptotic programs and thereby driving PANoptosis-like cell death.

A principal early crosstalk axis centers on Piezo1-mediated calcium influx and subsequent caspase-8 activation. Via this pathway, a single mechanosensitive stimulus can concomitantly activate multiple death programs: caspase-dependent apoptosis, RIPK3/MLKL-mediated necroptosis, and NLRP3/caspase-1/GSDMD-driven pyroptosis [43]. This convergence underscores that early I/R injury represents not merely the coexistence of distinct PCD modalities, but a highly coordinated network response orchestrated around shared upstream mechanosensing.

#### 3.1.2 Late Phase Dominance: Ferroptosis Cascade Activation and Key Regulatory Pathways

As injury progresses, the network transitions toward ferroptosis predominance. In the later phase, collapse of antioxidant defenses, accumulation of lipid peroxidation products, and GPX4 destabilization constitute a second major pathogenic hub [44,45,46,47]. Consequently, the overall architecture of myocardial I/R injury can be conceptualized as an early phase dominated by mechanically triggered and inflammatory cell death, followed by a subsequent ferroptosis-amplification stage.

A second major crosstalk axis arises in the later phase through redox imbalance and lipid metabolic dysregulation. GPX4 serves as a central ferroptosis checkpoint; its destabilization—via 4-HNE-mediated ubiquitination or autophagic degradation—exacerbates lipid peroxide accumulation and drives cardiomyocyte demise [44]. Additional modulators, including OTUD5, the circular RNA Nicotinamide Phosphoribosyltransferase/Sirtuin 1/Forkhead box protein O1/Ferritin Heavy Chain 1 (circRNA-NAMPT/SIRT1/FOXO1/FTH1) axis, and the ALOX15/15-HpETE pathway, further regulate ferroptosis susceptibility [45,46]. Critically, these ferroptotic processes are not isolated from earlier events: mitochondrial injury and ROS produced during the acute inflammatory phase likely sensitize cells to subsequent redox collapse.

#### 3.1.3 Bidirectional Autophagic Regulation Throughout the Pathogenesis

Autophagy constitutes a third axis that spans the entire course of I/R injury. Adequate autophagic flux facilitates clearance of damaged mitochondria and oxidized components, thereby attenuating ROS accumulation and suppressing both apoptotic and ferroptotic injury [48,49]. Conversely, impaired autophagy—including disruption of Atg7-dependent processes—aggravates inflammation, mitochondrial injury, and cardiomyocyte loss [50,51]. Thus, rather than operating as an independent parallel pathway, autophagy functions primarily as a context-dependent modulator that dictates whether the overall PCD network is attenuated or amplified.

Collectively, myocardial I/R injury is best conceptualized as a stage-dependent PCD network, wherein the dominant cell death phenotype transitions from early PANoptosis-like injury to later ferroptosis-driven execution, with autophagy serving as a key modulator of overall injury progression. This framework carries significant therapeutic implications: interventions likely need to be stage-specific rather than uniformly administered throughout reperfusion. Early-phase strategies may prove more efficacious when targeting mechanotransduction and inflammatory death coordination, whereas late-phase approaches should focus on inhibiting lipid peroxidation and restoring antioxidant capacity. Critical knowledge gaps persist, including the precise temporal sequence of pathway activation in human myocardium, the extent of cell-type specificity among cardiomyocytes and non-cardiomyocytes, and the identification of biomarkers capable of distinguishing early network configurations from late ferroptotic dominance.

### 3.2 HF and Cardiomyopathy: Regulatory Mechanisms of PCD

Heart failure and cardiomyopathies constitute convergent clinical endpoints of varied cardiovascular insults, yet the organization of the PCD network exhibits distinct etiology-dependent patterns. In certain cardiomyopathies, mitochondrial redox stress serves as a central integrative hub coordinating multiple death programs, whereas in others, ferroptosis predominates as the primary effector within a broader hub-centered network. The subsequent sections delineate these disease-specific configurations.

#### 3.2.1 DOX-Induced Cardiomyopathy: A Synergistic Multi-PCD Injury Network

DOX-Induced Cardiomyopathy (DIC) represents a major dose-limiting adverse effect of anthracycline chemotherapy, characterized by progressive cardiomyocyte loss, myocardial fibrosis, and ventricular dysfunction [52]. Its pathogenesis cannot be fully accounted for by any single PCD modality. Instead, DIC exemplifies a multilayered PCD network orchestrated by sustained oxidative stress, mitochondrial injury, inflammatory activation, and dysregulated iron homeostasis.

##### 3.2.1.1 Mitochondrial ROS as the Central Integrative Hub

Among these stressors, mitochondrial ROS constitute the primary integrative hub. Mitochondrial ROS interconnects iron-dependent oxidative damage, apoptotic mitochondrial permeabilization, inflammasome-driven injury, and autophagic dysregulation [53]. Within this network, ferroptosis emerges as the predominant pathogenic effector, as doxorubicin disrupts both iron metabolism and lipid peroxide detoxification, with its impact further amplified by concurrent apoptotic and inflammatory pathways rather than acting in isolation.

##### 3.2.1.2 Ferroptosis-Centric Oxidative Axis

The most prominent crosstalk axis in DIC is the ferroptosis-centric oxidative module. Enhanced TFRC-mediated iron uptake, mitochondrial glutathione depletion, GPX4 destabilization, and impaired iron export collectively fuel cardiomyocyte lipid peroxidation [47,54,55,56,57]. Multiple regulators—including sorting nexin-3 (SNX3), FUN14 Domain Containing 2 (FUNDC2), ACSL4/ferritin heavy chain 1 (FTH1) signaling, and NEDD4L-dependent GPX4 ubiquitination—contribute to this cascade, while restoration of iron export confers protection [47,54,55,56,57].

##### 3.2.1.3 Immune–Ferroptosis Axis

A second key axis links inflammatory signaling to enhanced ferroptosis susceptibility. IL-12p40-dependent immune activation has been demonstrated to exacerbate myocardial ferroptosis, revealing that innate immune responses and iron-dependent oxidative cell death are mechanistically interdependent rather than simply coexistent [58].

##### 3.2.1.4 Mitochondrial Dysfunction, Apoptosis, and Pyroptosis

A third axis interconnects mitochondrial dysfunction with apoptosis and pyroptosis. In DIC, classical apoptosis is mediated by Bax/Bcl-2 dysregulation, cytochrome c release, and subsequent caspase activation [7,59]. Pyroptosis contributes through NLRP3 inflammasome activation, leading to gasdermin-dependent membrane permeabilization and release of pro-inflammatory cytokines [60]. These pathways share key upstream triggers with ferroptosis—most notably ROS accumulation and mitochondrial injury—explaining why inhibition of any single downstream death route typically provides only partial protection.

##### 3.2.1.5 Autophagy as a Modulatory Layer

Autophagy constitutes an additional regulatory layer. When properly maintained, it promotes clearance of damaged mitochondria and mitigates oxidative stress. Conversely, dysregulated autophagic flux can exacerbate cardiomyocyte loss and amplify apoptotic or pyroptotic injury [60,61,62]. Regulatory modules, including the miR-34a-5p/Sirtuin 3 (SIRT3)/AMP-activated protein kinase (AMPK) axis and high mobility group box 1 protein-dependent protein kinase B/mammalian target of rapamycin (HMGB1-dependent Akt/mTOR) signaling, modulate this balance [61,62], reinforcing that DIC arises from network-level dysregulation rather than isolated pathway activation.

##### 3.2.1.6 Conceptual Implications and Open Questions

Collectively, DIC is best conceptualized as a hub-centered PCD network in which mitochondrial ROS orchestrates ferroptosis-predominant injury in concert with apoptotic, pyroptotic, and autophagy-related dysfunction. This paradigm implies that therapeutic strategies targeting shared upstream nodes, mitochondrial preservation, or coordinated pathway modulation may afford more comprehensive cardioprotection than single-pathway inhibition alone. Major unresolved questions include the temporal sequence of these death programs, the precise mechanisms linking immune activation to ferroptosis sensitization, and the development of interventions that preserve antitumor efficacy.

#### 3.2.2 DCM: A Ferroptosis-Centric PCD Network With Mitochondrial Dysfunction as the Hub

Diabetic cardiomyopathy (DCM) represents a major complication of type 2 diabetes mellitus, characterized by progressive cardiomyocyte loss, mitochondrial dysfunction, myocardial fibrosis, and adverse ventricular remodeling, ultimately culminating in heart failure [63]. In contrast to DIC, where multiple PCD modalities are more evenly integrated, DCM exhibits a more distinctly ferroptosis-centric architecture driven by chronic metabolic stress.

**Fig. 1.**
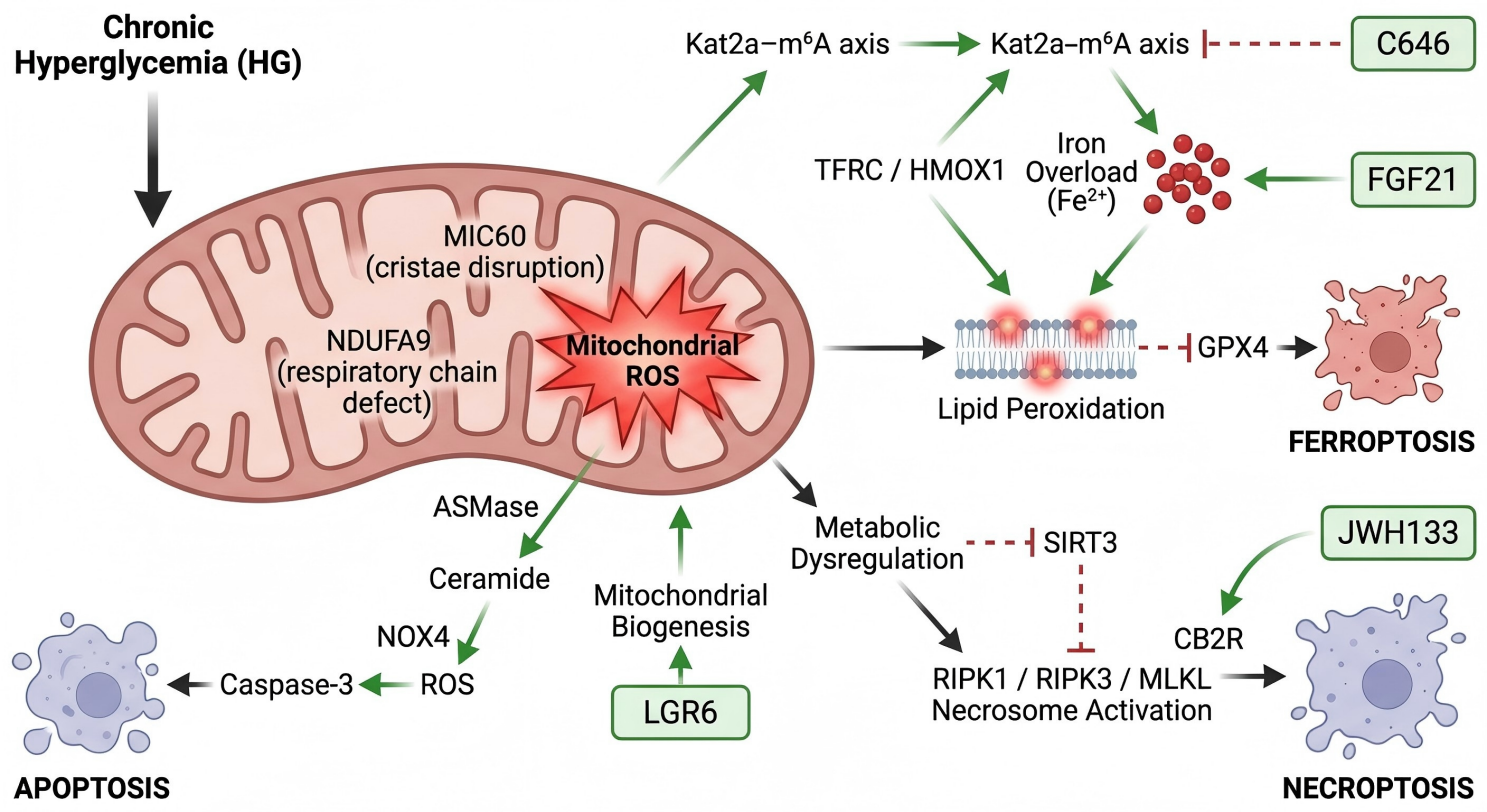
**Mitochondrial dysfunction-centered programmed cell death network in diabetic cardiomyopathy**. Chronic hyperglycemia acts as a persistent metabolic stressor that converges on mitochondria, leading to structural disruption and respiratory chain dysfunction. Impaired mitochondria serve as a central signaling hub by generating excessive ROS, which coordinate multiple forms of programmed cell death. Ferroptosis represents the dominant pathway, driven by epigenetic dysregulation of iron metabolism and suppression of lipid peroxide detoxification, ultimately resulting in iron-dependent lipid peroxidation. In parallel, mitochondrial ROS promotes apoptosis through lipid-mediated oxidative signaling and activates necroptosis via metabolic and redox-sensitive necrosome formation. These pathways are not isolated but interconnected through shared mitochondrial signals. Regulatory and pharmacological intervention nodes target distinct components of this network, converging on mitochondrial protection, redox balance, and iron homeostasis to mitigate cardiomyocyte loss in diabetic cardiomyopathy. Abbreviations: HG, hyperglycemia; ROS, reactive oxygen species; TFRC, transferrin receptor 1; HMOX1, heme oxygenase 1; GPX4, glutathione peroxidase 4; ASMase, acid sphingomyelinase; NOX4, NADPH oxidase 4; RIPK, receptor-interacting protein kinase; MLKL, mixed lineage kinase domain-like protein; CB2R, cannabinoid receptor 2. This figure was created by BioRender.

As depicted in Fig. 1, chronic hyperglycemia converges on mitochondrial dysfunction, which functions as the primary signaling hub orchestrating multiple PCD outputs in DCM. Within this network, ferroptosis constitutes the predominant pathway, as sustained hyperglycemic stress impairs iron homeostasis, compromises antioxidant defenses, and perpetuates lipid peroxidation [63,64,65,66]. Thus, the defining architecture of DCM is not merely “high glucose induces ferroptosis,” but rather “chronic metabolic stress inflicts mitochondrial damage, which in turn organizes a ferroptosis-centered death network”.

The most prominent axis is the ferroptosis module itself. Sustained hyperglycemia promotes iron uptake and mobilization, disrupts ferritin-mediated iron buffering, and impairs antioxidant capacity, thereby sensitizing cardiomyocytes to lipid peroxidation-driven death [63,64,65,66]. Fibroblast growth factor 21 (FGF21) attenuates this process by maintaining ferritin-associated iron sequestration, while leucine-rich repeat-containing G protein-coupled receptor 6 (LGR6)-dependent mitochondrial biogenesis alleviates oxidative stress and reduces ferroptosis susceptibility [63,64,65]. Furthermore, the lysine acetyltransferase 2 A (KAT2A)–m6A axis connects epigenetic dysregulation to upregulated expression of iron-related genes (e.g., TFRC and HMOX1), thereby reinforcing ferroptosis [66].

A second axis is mediated by mitochondrial ROS, which bridges ferroptosis with other death pathways. Structural mitochondrial disruption, cristae disorganization, and respiratory chain impairment elevate ROS production, which accelerates lipid peroxidation while simultaneously promoting apoptotic signaling and necroptotic activation [65,66,67,68,69,70]. As clearly illustrated in Fig. 1, mitochondrial failure constitutes both a pathological consequence of hyperglycemia and a central decision-making platform that governs downstream PCD selection.

A third axis encompasses the auxiliary roles of apoptosis and necroptosis. In DCM, apoptosis is driven by mitochondrial membrane damage and oxidative signaling, exemplified by the ASMase (acid sphingomyelinase)/NOX4 (NADPH oxidase 4) pathway and the TRAP1 (TNF receptor-associated protein 1)–MARCH 5 (membrane-associated RING-CH protein 5)–MIC60 (mitofilin) axis, both of which compromise mitochondrial integrity and trigger caspase-dependent death [67,68]. Necroptosis is driven by redox-sensitive inflammatory signaling via the RIPK1/RIPK3/MLKL cascade, exacerbated by loss of CB2R-mediated suppression and SIRT3 deficiency [69,70]. These pathways do not supplant ferroptosis as the primary effector but rather amplify myocardial inflammation and adverse remodeling within the shared stress network.

Collectively, DCM is best conceptualized as a ferroptosis-centered yet mitochondria-orchestrated PCD network. This framework elucidates why interventions that concurrently restore mitochondrial homeostasis, reestablish redox balance, and suppress ferroptotic signaling are likely to outperform strategies targeting individual downstream pathways. Significant knowledge gaps persist concerning the roles of non-cardiomyocyte cell types, the influence of heterogeneous diabetic phenotypes and comorbidities, and the long-term safety of ferroptosis-targeted therapies in chronic metabolic disease.

#### 3.2.3 Pressure-Overload HF: An Apoptosis-Autophagy Synergistic PCD Network

Pressure-overload heart failure (PO-HF), commonly triggered by chronic hypertension or experimental aortic constriction, exemplifies a classic form of mechanical stress-induced cardiomyopathy. It is characterized by progressive cardiomyocyte hypertrophy, interstitial fibrosis, and eventual adverse ventricular remodeling. In this context, the PCD network is predominantly driven by synergistic apoptosis–autophagy interactions, exhibiting a distinct organizational pattern compared with that observed in metabolic(DCM) or toxic (DIC) settings.

A pivotal regulatory hub in pressure-overload heart failure is the transcription factor E26 transformation specific 2 (ETS2), which orchestrates apoptosis–autophagy synergy. ETS2 directly binds the ETS motif in the promoter of the long non-coding RNA taurine upregulated gene 1 (TUG1), thereby promoting its transcription. As a competing endogenous RNA (ceRNA), TUG1 sequesters miR-129-5p, relieving its suppression of autophagy related 7 (ATG7). The resulting upregulation of ATG7 enhances autophagosome formation (evidenced by increased LC3-II levels) while concurrently promoting caspase-3 activation, ultimately driving apoptotic execution [71]. Genetic knockout of ETS2 substantially reduces both excessive autophagy and apoptosis, preserving cardiac contractility and confirming the ETS2–TUG1–miR-129-5p–ATG7 axis as a central pro-death regulatory pathway.

Beyond this transcriptional regulatory axis, bioactive constituents of Si-Miao-Yong-An Decoction—namely Angoroside C (AC) and 3,5-dicaffeoylquinic acid (3,5-DiCQA)—modulate the PDE5A–Akt and TLR4–NOX4 signaling pathways. Pressure overload or isoproterenol stimulation activates PDE5A (suppressing Akt signaling) and engages the TLR4–NOX4 axis (elevating reactive oxygen species production), collectively driving excessive autophagy and Bax-dependent apoptosis. AC and 3,5-DiCQA inhibit PDE5A, restore Akt activity, and suppress TLR4–NOX4-mediated oxidative stress, thereby attenuating both processes [72]. Co-administration of the autophagy inhibitor 3-methyladenine further diminishes apoptosis, supporting the notion that excessive autophagy functions as an upstream amplifier of apoptotic execution within this synergistic network.

**Fig. 2.**
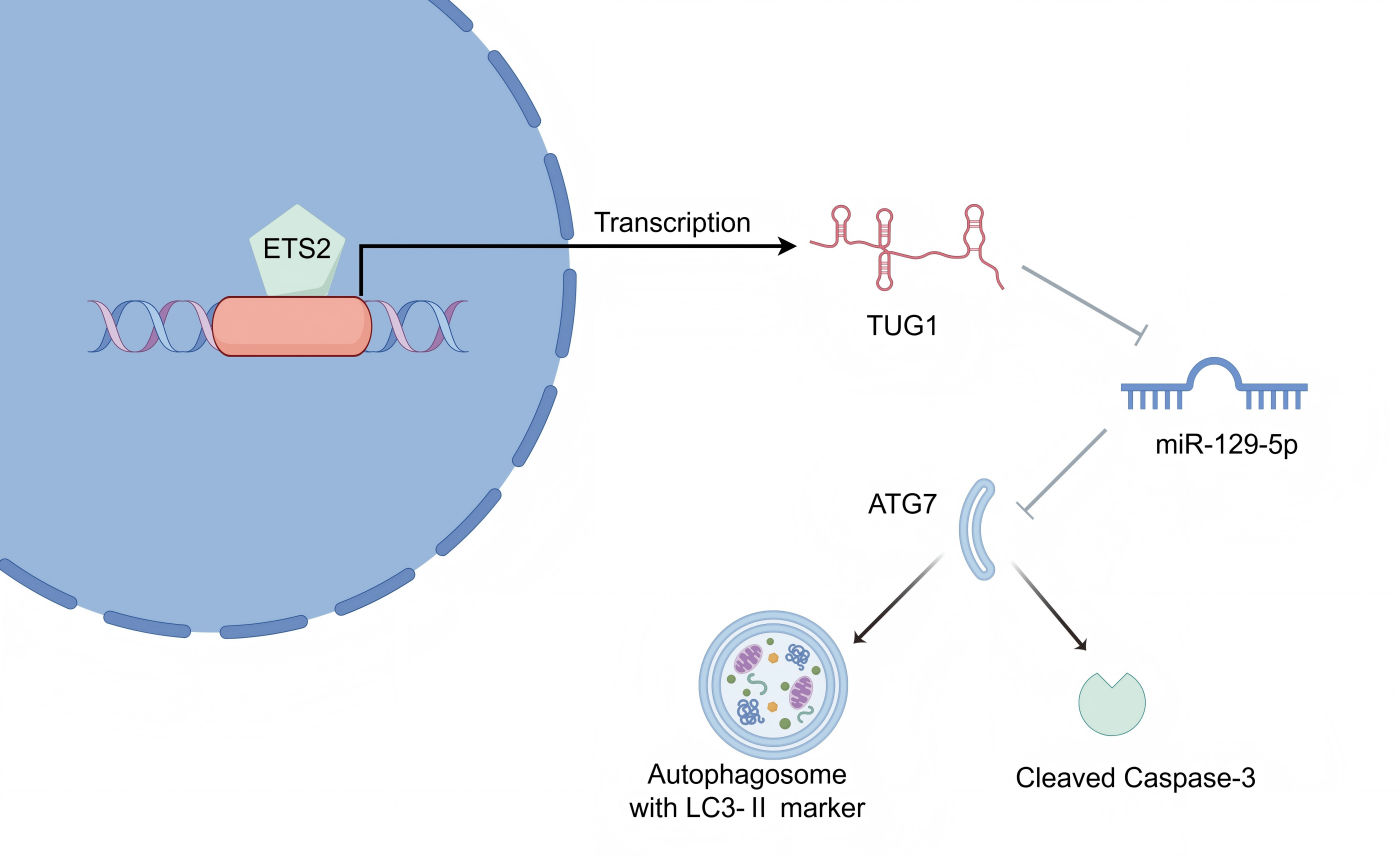
**Schematic illustration of the key molecular mechanisms underlying the synergistic interplay between apoptosis and autophagy in pressure overload–induced heart failure**. In cardiomyocyte nuclei, the transcription factor ETS2 binds to the promoter regions of its target genes, thereby enhancing the transcriptional expression of the long non-coding RNA TUG1. Upregulated TUG1 acts as a competing endogenous RNA for miR-129-5p, attenuating its inhibitory effect on the autophagy-related protein ATG7. This derepression of ATG7 promotes autophagic activity (evidenced by LC3-II conversion and autophagosome formation) and simultaneously triggers apoptotic signaling, leading to the cleavage of caspase-3 from its inactive precursor to the active form. Collectively, the ETS2–TUG1–miR-129-5p–ATG7 axis constitutes a central regulatory network mediating the coupled dysregulation of apoptosis and autophagy in cardiomyocytes, providing a crucial molecular basis for pathological remodeling during pressure overload–induced heart failure. ETS2, E26 transformation specific 2; TUG1, taurine upregulated gene 1; ATG7, autophagy related 7. This figure was created by Figdraw 2.0.

Synergistic apoptosis–autophagy activation also establishes a self-reinforcing vicious cycle with myocardial fibrosis: apoptotic and autophagic cardiomyocyte loss stimulates cardiac fibroblast activation and excessive collagen deposition; resultant fibrosis compromises myocardial perfusion and oxygenation, thereby perpetuating PCD signaling. Targeting key hub nodes—such as ETS2 or oxidative stress-related pathways—simultaneously attenuates PCD activation and limits fibrotic progression, offering dual therapeutic benefits. The sequential relationship among PCD activation, cardiomyocyte loss, and fibrosis amplification under pressure‑overload conditions is illustrated in Fig. 2. Nevertheless, significant knowledge gaps persist, most notably the precise molecular mechanisms linking profibrotic cytokines to PCD pathway activation, the potential integration of necroptosis and pyroptosis into this network, and the absence of robust clinical validation for ETS2 inhibitors or TLR4 antagonists in humans.

### 3.3 Cell-Specific Regulation and Clinical Value of the PCD Network in AS

In AS, the PCD network is organized predominantly according to cell type rather than through a single universal pathway. Endothelial cells, macrophages, and vascular smooth muscle cells (VSMCs) exhibit distinct dominant death signatures, with the pathological outcomes of these signatures governed by their dynamic interplay within the plaque microenvironment. This cell–type–resolved network perspective provides a more comprehensive framework for understanding plaque progression than models centered on any individual death pathway [73].

#### 3.3.1 Vascular Endothelial Cells: Dual Drivers of Plaque Initiation Mediated by Pyroptosis and Ferroptosis

Endothelial dysfunction constitutes an early and pivotal event in AS. As endothelial cells form the critical interface between circulating lipids, inflammatory mediators, and the vascular wall, their injury rapidly compromises barrier integrity, promotes leukocyte recruitment, and facilitates lipid infiltration [74].

Mounting evidence suggests that endothelial injury in AS is predominantly driven by two coordinated PCD modalities: pyroptosis and ferroptosis. Rather than functioning redundantly, these pathways mediate complementary facets of endothelial dysfunction—pyroptosis primarily driving inflammatory disruption and ferroptosis accentuating oxidative membrane damage.

A primary axis involves oxidized low-density lipoprotein (ox-LDL)–induced pyroptotic injury. In endothelial cells, ox-LDL triggers caspase-3-mediated cleavage of gasdermin E (GSDME), resulting in pore formation, membrane rupture, release of inflammatory cytokines, and disruption of endothelial barrier integrity [75,76]. This process not only directly injures the endothelium but also promotes macrophage recruitment and transendothelial lipid deposition, thereby accelerating atherosclerotic lesion initiation.

A second key axis centers on endothelial ferroptosis triggered by metabolic disruption of antioxidant homeostasis. N-acetylneuraminic acid (Neu5Ac) promotes SLC3A2 degradation, thereby restricting cystine availability for glutathione synthesis, impairing GPX4-mediated lipid peroxide detoxification, and ultimately precipitating ferroptosis [77]. *In vivo*, inhibition of this axis attenuates endothelial ferroptosis, vascular inflammation, and plaque progression [77]. Functionally, this mechanism complements pyroptosis: whereas pyroptosis creates an inflammatory breach, ferroptosis exacerbates oxidative endothelial injury.

Collectively, endothelial pyroptosis and ferroptosis constitute a dual initiation module that drives atherosclerosis progression via synergistic effects on barrier disruption, inflammatory cell recruitment, and enhanced lipid infiltration. Their convergence positions endothelial cells as a compelling early therapeutic target. Nevertheless, the shared upstream regulators that couple these two pathways remain incompletely characterized, and robust clinical evidence supporting pathway-selective endothelial interventions is still scarce.

#### 3.3.2 Macrophages: Core Regulators of Plaque Stability Governed by PCD Modalities

Macrophages constitute the predominant immune effector cells within atherosclerotic plaques and play a pivotal role in foam cell formation, inflammatory amplification, necrotic core expansion, and plaque destabilization. More than any other cell type in atherosclerosis, macrophages exemplify the networked nature of PCD, as they can execute distinct death programs with markedly divergent consequences for plaque biology.

In macrophages, the critical “hub” is not a single molecular pathway but the macrophage itself, functioning as a key decision-making cellular node within the plaque microenvironment. The resulting death phenotype dictates whether plaque progression favors resolution, chronic persistence, or rupture-prone instability.

The first axis involves apoptosis, which exerts context-dependent effects. Moderate macrophage apoptosis can be beneficial, promoting efferocytosis of foam cells and restricting lipid accumulation, whereas excessive apoptosis drives necrotic core expansion and compromises plaque stability [78,79]. Natriuretic peptide receptor C (NPRC) deficiency confers protection, at least partly, by attenuating oxidative stress, limiting excessive macrophage apoptosis, and ameliorating inflammation [78].

A second key axis centers on macrophage ferroptosis, which markedly promotes plaque vulnerability. Dysregulated erythrophagocytosis and intracellular iron overload—observed, for example, in erythroid Jak2V617F contexts—drive ROS generation, lipid peroxidation, and ferroptotic death [80]. Ferroptotic macrophages release DAMPs that amplify local inflammation and accelerate necrotic core expansion. The Kelch-like ECH-associated protein 1–nuclear factor erythroid 2-related factor 2 (KEAP1–Nrf2) axis further regulates this process by modulating expression of key antioxidant genes, including GPX4 and heme oxygenase-1 (HO-1) [81]. Human plaque analyses corroborate the clinical relevance of ferroptosis, showing that Prostaglandin-endoperoxide synthase 2 (PTGS2) expression correlates with inflammatory burden and lipid-rich plaque characteristics [82].

A third axis involves macrophage pyroptosis, which directly links innate immune activation to plaque instability. Piezo1-mediated calcium influx activates the NLRP3 inflammasome, resulting in caspase-1 activation, GSDMD cleavage, release of inflammatory cytokines, and foam cell injury [83,84]. This cascade undermines plaque stability not only by amplifying inflammation but also by impairing vascular smooth muscle cell (VSMC)–dependent fibrous cap integrity. Pharmacological modulation of the Piezo1–NLRP3–GSDMD axis (e.g., with Guizhitongluo Tablet) further underscores the significance of this pathway in plaque progression [83].

Collectively, macrophage PCD serves as the primary switch governing plaque stability. Apoptosis can exert adaptive or detrimental effects depending on its extent and efferocytosis efficiency, whereas ferroptosis and pyroptosis predominantly promote vulnerability by amplifying inflammation and necrotic progression. Future research should prioritize subtype- and stage-specific modulation of macrophage death programs, elucidation of PCD heterogeneity across macrophage subsets, and validation of biomarkers such as PTGS2 for clinical risk stratification.

#### 3.3.3 Vascular Smooth Muscle Cells: Regulation of Mechanical Stability by Apoptosis-Mediated Fibrous Cap Thinning

VSMCs represent the primary structural cells responsible for preserving fibrous cap thickness and mechanical integrity. Their depletion exerts direct biomechanical consequences, significantly increasing plaque vulnerability.

Among currently characterized pathways, apoptosis constitutes the predominant mode of VSMC death associated with plaque destabilization. Although other PCD modalities may also contribute, the most robustly supported pathogenic mechanism in this cell type remains apoptotic loss of cap-forming VSMCs.

Inflammatory cytokines, notably IL-1β and TNF-α, within the plaque microenvironment directly trigger VSMC apoptotic signaling [85,86]. Progressive VSMC loss reduces collagen synthesis, resulting in fibrous cap thinning and diminished mechanical strength. Concurrently, cellular debris and inflammatory mediators released from the unstable plaque exacerbate macrophage ferroptosis and pyroptosis, establishing a vicious intercellular cycle: inflammation drives VSMC apoptosis, VSMC depletion compromises cap integrity, and escalating plaque vulnerability ensues [83,84,86,87].

Hence, VSMC apoptosis should be regarded not as an isolated structural event, but as an integral component of an intercellular PCD network that couples inflammatory macrophage death to mechanical plaque destabilization. Nevertheless, mechanistic insights into VSMC PCD remain less advanced compared with those for endothelial cells or macrophages. Key unresolved questions include: how phenotypic switching between contractile and synthetic VSMC states modulates apoptotic susceptibility; whether non-apoptotic PCD modalities contribute substantially to VSMC depletion; and how these pathways dynamically interact throughout plaque progression.

Collectively, the PCD network in atherosclerosis is best conceptualized as an intercellular system: endothelial pyroptosis and ferroptosis initiate lesion formation, macrophage multimodal PCD drives inflammatory amplification and plaque vulnerability, and VSMC apoptosis governs fibrous cap integrity. This cell–type–specific organization accounts for why plaque progression cannot be ascribed to any single death pathway and underscores the necessity for future interventions to target both the predominant cell type and the dominant PCD modality within the evolving plaque microenvironment.

## 4. Therapeutic Opportunities and Translational Challenges of Targeting the PCD Network

The PCD network in CVDs offers promising therapeutic avenues, with preclinical studies showing that targeting key pathways or shared regulatory nodes—including via natural compounds and selective inhibitors—can effectively protect against myocardial I/R injury, heart failure, and atherosclerosis. However, successful clinical translation still hinges on overcoming major barriers such as tissue specificity, safety concerns, and the lack of reliable pathway-specific biomarkers.

### 4.1 Targeted Intervention Strategies for Specific PCD Pathways

Aberrant activation of distinct PCD pathways plays a pivotal role across different stages of CVDs. Pathway-specific inhibitors that selectively target core molecular nodes of detrimental PCD signaling have shown robust cardioprotective effects in preclinical models. Table 1 (Refs. [16,17,24,48,88,89,90,91,92,93,94,95,96]) systematically summarizes the therapeutic potential of representative pathway-specific agents, detailing their molecular targets, preclinical efficacy, key pharmacological properties, and translational implications (Table 1).

**Table 1.** **Mechanism of PCD-targeted inhibitors in the main pathological changes of cardiovascular diseases**.

Pathological change	Animal/Cell	Model	Intervention (Representative drug)	Effect	Mechanism (Core molecular targets)	Ref
Ferroptosis-related damage	C57BL/6 mice	HFpEF	Liproxstatin-1	LV diastolic function ↑; myocardial fibrosis ↓	GPX4 preserved; FSP1–CoQ10 antioxidant axis enhanced	[94]
	Lung transplantation model	Cold I/R injury	Liproxstatin-1	Lung function recovery ↑; ROS ↓	GPX4 lipid peroxide clearance; FSP1 activation	[93]
	H9c2 cells	Myocardial I/R	Melatonin	Infarct size ↓; cell survival ↑	ATM/p53–IRP1–TFRC inhibited; AMPK/Nrf2–HO-1/SLC7A11/GPX4 activated	[92,95]
	C57BL/6 mice	Myocardial I/R	Isoliquiritigenin	4-HNE ↓; LVEF ↑	Keap1 oxidation → Nrf2 nuclear translocation → HO-1, SLC7A11, GPX4 ↑	[96]
Apoptosis-related damage	C57BL/6 mice	MI	Navitoclax (ABT-263)	Caspase-3/9 ↓; infarct size ↓	BCL-2 inhibition → Bax/Bak activation	[16]
	C57BL/6 mice	DIC	SM-164	Apoptosis ↓; LVFS ↑	XIAP inhibition → caspase-3/7 activation	[17]
Necroptosis-related damage	C57BL/6 mice	Myocardial I/R	Necrostatin-1	p-MLKL ↓; TNF-α ↓	RIPK1 kinase activity inhibited	[24,89]
	C57BL/6 mice	Myocardial I/R	GSK872 + Nec-1	Necroptosis inhibition ↑	RIPK3 autophosphorylation blocked → MLKL inhibition	[24,88]
Pyroptosis-related damage	C57BL/6 mice	PAH-induced right HF	MCC950	IL-1β/IL-18 ↑; RVEF ↑	NLRP3 inflammasome ATPase activity inhibited	[90,91]
Autophagy imbalance	C57BL/6 mice	Myocardial I/R	Sephin1	Autophagic flux restored; infarct size ↓	eIF2α-mediated ISR activation; autophagy–apoptosis balance	[48]

Abbreviations: HFpEF, heart failure with preserved ejection fraction; I/R, ischemia-reperfusion; MI, myocardial infarction; DIC, doxorubicin-induced cardiomyopathy; FSP1, ferroptosis suppressor protein 1; LVEF, left ventricular ejection fraction; LVFS, left ventricular fractional shortening; RVEF, right ventricular ejection fraction; PAH, pulmonary arterial hypertension; p-MLKL, phosphorylated mixed lineage kinase domain-like protein; XIAP, X-linked inhibitor of apoptosis protein. ↑: increase/upregulation; ↓: decrease/downregulation.

Selective inhibitors targeting individual PCD pathways—namely ferroptosis, necroptosis, pyroptosis, and autophagy—offer precise interventional tools for CVDs, with multiple agents showing robust preclinical efficacy.

Ferroptosis inhibitors, primarily targeting GPX4 and FSP1, exert robust cardioprotective effects in myocardial I/R injury, HF, and related conditions. Liproxstatin-1, a prototypical ferroptosis inhibitor, preserves GPX4 activity, suppresses lipid peroxidation, and markedly attenuates cold I/R injury in lung transplantation [93] as well as renal tubular ferroptosis and subsequent fibrosis in unilateral ureteral obstruction models [97]. In cardiovascular contexts, Liproxstatin-1 ameliorates the pathological phenotype in HF with preserved ejection fraction (HFpEF) mouse models [94], suggesting a promising therapeutic avenue for this challenging HF subtype. Furthermore, natural compounds such as melatonin mitigate ferroptosis in myocardial I/R injury by suppressing (ATM)/p53 signaling [95], whereas isoliquiritigenin confers cardioprotection by activating the Nrf2/HO-1/SLC7A11/GPX4 axis [96]. Similarly, 3-hydroxyanthranilic acid analogs attenuate I/R-induced myocardial ferroptosis via the same pathway [98], collectively highlighting the broad therapeutic potential of ferroptosis inhibition across diverse CVD contexts.

Necroptosis inhibitors primarily target the RIPK1/RIPK3/MLKL axis. Necrostatin-1 (Nec-1), a selective RIPK1 inhibitor, has demonstrated efficacy across multiple disease models. In combination with GSK872, Nec-1 protects retinal ganglion cells from necroptosis in glutamate-induced glaucoma by preventing necrosome assembly and attenuating proinflammatory cytokine release [88,89].

Pyroptosis inhibitors primarily target the NLRP3 inflammasome and its downstream effectors. MCC950, a potent and selective NLRP3 inhibitor, has shown efficacy in various inflammation-driven disorders [90]. Notably, NLRP3 activation in macrophages is a major driver of right ventricular failure in pulmonary arterial hypertension, and its inhibition substantially improves right ventricular function [91], underscoring the therapeutic promise of pyroptosis inhibitors in cardiopulmonary pathologies.

Given the “double-edged” nature of autophagy, precise modulation of autophagic flux is essential. In myocardial I/R injury, moderate autophagy facilitates the clearance of damaged mitochondria, whereas excessive autophagic activity aggravates myocardial injury. Melatonin activates the AMPK/Nrf2 pathway to promote adaptive autophagy, thereby clearing oxidative damage products and suppressing apoptosis, ultimately conferring cardioprotection [92]. Similarly, Sephin1 potentiates the integrated stress response (ISR) to induce controlled autophagy and inhibit apoptosis, significantly reducing infarct size in myocardial I/R models [48], reinforcing the pivotal role of autophagy modulators in fine-tuning cell survival versus death decisions.

### 4.2 Multi-Target Drugs for Crosstalk Nodes: Innovative Mechanisms and Pharmacological Advantages

Extensive crosstalk within the PCD network substantially limits the efficacy of single-pathway inhibitors. In many cardiovascular contexts, multiple PCD modalities are activated concurrently or sequentially, such that inhibition of one terminal pathway often leaves parallel detrimental routes unaffected. This constraint has heightened interest in agents targeting shared upstream regulators or key crosstalk nodes, thereby simultaneously modulating multiple death programs.

Piezo1 exemplifies a key crosstalk node. As a mechanosensitive ion channel, it transduces biomechanical stress into calcium influx, mitochondrial dysfunction, and subsequent activation of inflammatory and cell death signaling [43]. Experimental evidence demonstrates that Piezo1 inhibition mitigates overlapping features of apoptosis, necroptosis, and pyroptosis in myocardial I/R injury, reinforcing the notion that upstream mechanotransduction orchestrates multiple PCD modalities [43,99]. The peptide inhibitor GsMTx4 (Grammostola spatulata mechanotoxin 4) has demonstrated cardioprotective effects in preclinical models, further underscoring the therapeutic value of targeting shared proximal signaling nodes rather than isolated terminal effectors [100,101,102,103].

Nrf2 constitutes another highly attractive multi-target regulator. Rather than modulating a single death pathway, Nrf2 broadly augments antioxidant and cytoprotective defenses by upregulating genes involved in redox homeostasis, glutathione biosynthesis, and lipid peroxide detoxification [96,98]. Consequently, Nrf2 activation suppresses ferroptosis while concurrently attenuating secondary apoptosis and inflammatory responses. This multifaceted protective profile is likely to be particularly advantageous in settings where oxidative stress acts as a shared upstream driver of multiple PCD pathways.

Natural compounds further exemplify the potential of multi-target modulation. Melatonin, for instance, confers protection across multiple PCD-related processes by mitigating oxidative stress, maintaining mitochondrial homeostasis, and suppressing inflammatory signaling [60,92,95]. Its pleiotropic actions extend beyond any single pathway, positioning melatonin as a valuable prototype for network-oriented intervention in CVDs. More broadly, the pharmacological attractiveness of such agents stems from their capacity to modulate convergent regulatory nodes rather than merely terminal executioners.

Collectively, the principal advantage of multi-target agents lies in their capacity to address the integrated and redundant nature of the PCD network. Nevertheless, successful clinical translation will hinge on achieving adequate selectivity, optimal timing, and precise tissue distribution to prevent unintended disruption of physiological cell turnover or adaptive stress responses.

### 4.3 Key Challenges and Future Innovative Directions in Clinical Translation

Despite considerable preclinical advances, substantial barriers persist in translating PCD-targeted therapies from bench to bedside, highlighting the necessity for both technological innovation and strategic refinement.

A primary challenge is non-specific tissue distribution. For instance, although Liproxstatin-1 effectively suppresses myocardial ferroptosis, systemic administration may inadvertently alter ferroptosis susceptibility in non-cardiac tissues, potentially eliciting off-target effects [104]. Future efforts should focus on developing cardiomyocyte-specific delivery platforms—such as lipid nanoparticles functionalized to recognize surface markers enriched in injured myocardium—to enhance myocardial targeting while minimizing off-target accumulation in the liver, kidneys, and other organs.

Another critical translational concern pertains to the safety profile of multi-target inhibitors. As apoptosis, autophagy, pyroptosis, necroptosis, and ferroptosis also play essential roles in physiological tissue homeostasis, immune surveillance, and clearance of damaged cells, concurrent suppression of multiple PCD pathways carries the risk of unintended adverse consequences [7,8]. Potential risks encompass impaired clearance of dysfunctional cardiomyocytes or vascular cells, disruption of adaptive stress responses (e.g., basal autophagy), attenuation of protective inflammatory signaling, and off-target toxicity in non-cardiac organs. These concerns are particularly pertinent in cardiovascular contexts, where shared upstream regulators—such as ROS, mitochondrial dysfunction, and inflammatory mediators—can mediate either pathological injury or compensatory adaptation depending on cellular context. Moreover, broad inhibition of ferroptosis- or apoptosis-related pathways could theoretically affect tumor biology or therapeutic responsiveness in patients with coexisting malignancies [5,104]. Consequently, the therapeutic index of multi-target agents may be narrower than anticipated, rendering dose optimization, disease-stage alignment, and tissue-specific delivery critical for safe and effective clinical translation.

A further key consideration is the pronounced temporal dependence of PCD network activation. In myocardial I/R injury, for example, Piezo1-mediated PANoptosis predominates in the early phase, whereas ferroptosis becomes more prominent in later stages [43,44]. This temporal shift underscores the need for stage-specific therapeutic strategies rather than uniform application. Early-phase interventions should prioritize blockade of stress-induced inflammatory death programs, whereas later-phase interventions may be more efficacious when directed at lipid peroxidation and mitochondrial redox dysregulation.

PCD modalities exhibit considerable cell-type specificity. In atherosclerosis, endothelial cells predominantly undergo pyroptosis and ferroptosis, whereas macrophages display a broader spectrum encompassing apoptosis, ferroptosis, and pyroptosis [77,80,83]. Single-cell RNA sequencing and complementary multi-omics approaches hold promise for delineating cell-type-specific PCD signatures, identifying dominant pathogenic cell populations across disease contexts, and informing the design of more precise therapeutic strategies [105].

Currently, the majority of PCD-targeted agents remain at the preclinical stage, with only a small number of related strategies having advanced to clinical evaluation [90]. Existing clinical trials have primarily targeted inflammatory pathways rather than directly addressing specific PCD mechanisms. Future translational efforts should therefore prioritize the progression of promising candidates into early-phase clinical trials, while concurrently developing pathway-specific biomarkers to enable patient stratification and treatment response monitoring. Candidate biomarkers, such as PTGS2 for ferroptosis and phosphorylated MLKL (p-MLKL) for necroptosis, may enhance precision in trial design and therapeutic efficacy assessment [82].

## 5. Limitations

Despite the expanding body of evidence synthesized in this review, several important limitations warrant acknowledgment.

First, the majority of insights into PCD networks in cardiovascular diseases derive from *in vitro* studies and animal models, with direct validation in human myocardial or vascular tissues remaining scarce. Consequently, certain mechanistic inferences may lack full translatability across species or clinical disease contexts.

Second, the relative contribution of individual PCD pathways exhibits strong dependence on cell type, disease stage, and local microenvironmental cues. Identical upstream stressors can elicit distinct death programs in cardiomyocytes, endothelial cells, macrophages, or vascular smooth muscle cells, with the predominant pathway often shifting dynamically during disease progression. This pronounced context dependence precludes the establishment of a universal hierarchy of PCD events applicable to all cardiovascular diseases.

Third, while emerging concepts—including pathway switching, coordinated lytic death, and PANoptosis-like signaling—offer a valuable integrative framework, the mechanistic demarcation between apoptosis, pyroptosis, necroptosis, ferroptosis, and autophagy is often blurred. Overlapping molecular signatures and variability in experimental methodologies across studies frequently confound accurate assignment of the dominant cell death phenotype.

Fourth, robust and validated biomarkers for pathway-specific monitoring remain inadequate for routine clinical translation. Although candidates such as PTGS2 (for ferroptosis), phosphorylated MLKL (p-MLKL, for necroptosis), and inflammasome-related mediators hold promise, their specificity, reproducibility, and utility in patients with heterogeneous cardiovascular phenotypes necessitate further rigorous validation.

Finally, as this is a narrative review rather than a systematic review or meta-analysis, study selection—despite our efforts to achieve a balanced and contemporary synthesis—may have been influenced by prevailing research trends, the availability of cutting-edge mechanistic studies, and the prioritization of representative over exhaustive evidence.

## 6. Conclusions

PCD is increasingly recognized as an integrated network process that critically governs the initiation, progression, and clinical outcome of CVDs. Rather than functioning in isolation, apoptosis, pyroptosis, ferroptosis, necroptosis, and autophagy interact extensively via shared upstream stressors, convergent molecular hubs, and cell-type-specific execution modules. This network-centric perspective elucidates the heterogeneous pathological manifestations across CVD entities and explains the frequent inadequacy of single-pathway inhibition strategies.

Notably, while PCD-targeted interventions hold considerable translational promise, their clinical development faces formidable challenges, including non-specific tissue distribution, paucity of robust biomarkers, disease-stage and context-dependent heterogeneity, and risks associated with broad multi-target suppression. Accordingly, future research should shift from merely delineating individual pathways toward developing precision-oriented translational strategies. Key priorities include:

High-resolution mapping of PCD network dynamics across temporal, spatial, and cell-type dimensions using single-cell and spatial multi-omics technologies.

Selective targeting of central crosstalk nodes that coordinately regulate multiple death programs, rather than isolated terminal effectors.

Development of biomarker-guided, stage-specific therapeutic interventions to optimize patient stratification and intervention timing.

Advancement of tissue-specific delivery systems and rigorous safety profiling to maximize therapeutic efficacy while minimizing off-target and systemic toxicity.

Collectively, transitioning from a single-pathway paradigm to an integrated PCD network framework offers a more comprehensive and realistic basis for deciphering cardiovascular injury mechanisms and developing next-generation therapeutic strategies.
